# Emerging Scholars and Junior Faculty Present New Strategies for Student Engagement in Gerontology

**DOI:** 10.1093/geroni/igab046.384

**Published:** 2021-12-17

**Authors:** Sarah Hahn, Jennifer Kinney

## Abstract

With the rapid aging of the population, the need for gerontological educators to identify pedagogical strategies to increase interest and prepare students continues to grow. Innovative approaches and educational practices contribute greatly to student success in the gerontological classroom. Literature on gerontological pedagogy has shed light on the success of high-impact practices, creative assignments, pedagogical interventions, and even different course modalities when it comes to effectively delivering gerontological content and engaging students. Additionally, the Academy for Gerontology in Higher Education (AGHE) provides a wealth of suggestions for creating and implementing effective gerontology courses and assignments. However, while we are familiar with these practices, we are not familiar with how specific groups of academics, such as emerging scholars and junior faculty, are utilizing them. Emerging scholars and junior faculty experience several major transitions as they prepare for life in academia. To ensure that emerging scholars and junior faculty are well prepared, we need to continue to empower these individuals to foster growth. This can be done by highlighting how emerging scholars and junior faculty have met the goals of maximizing and optimizing student learning. As such, the purpose of this symposium is to examine innovative approaches used by emerging scholars and junior academics in the gerontological classroom that have optimized student learning. This includes presentations on strategies for team-based learning, using intersectionality as a theoretical lens, and two creative written assignments, The Gerontological Movie Database Review and Interview an Elder.

